# Protective Potential of an Autogenous Vaccine in an Aerogenous Model of *Escherichia coli* Infection in Broiler Breeders

**DOI:** 10.3390/vaccines9111233

**Published:** 2021-10-22

**Authors:** Sofie Kromann, Rikke Heidemann Olsen, Anders Miki Bojesen, Henrik Elvang Jensen, Ida Thøfner

**Affiliations:** 1 Department of Veterinary and Animal Sciences, Faculty of Health and Medical Sciences, University of Copenhagen, 1870 Frederiksberg, Denmark; cava@sund.ku.dk (R.H.O.); miki@sund.ku.dk (A.M.B.); elvang@sund.ku.dk (H.E.J.); icnt@sund.ku.dk (I.T.); 2 DanHatch Denmark A/S, Rugerivej 26, 9760 Vrå, Denmark

**Keywords:** APEC, colibacillosis, challenge study, poultry disease, disease prevention, bacterin vaccine

## Abstract

In poultry, *Escherichia coli* is a common cause of high-cost infections. Consequently, autogenous vaccines are often used despite limited and conflicting evidence on their effectiveness have been presented. The present study aimed to investigate the efficacy of a commonly used autogenous vaccine, previously deemed ineffective, in an aerosol model of colibacillosis. Methods: Broiler breeders (*n* = 47) were randomly allocated to one of four groups (vaccinated and unvaccinated birds receiving an autogenous vaccine or sterile saline intramuscularly) and challenged with either aerosolised *E. coli* or vehicle at 29 weeks of age. Two days following inoculation, the birds were euthanised, thoroughly necropsied, and samples for bacteriology and histopathology were collected. Results: Vaccinated birds had a significantly lower bacteriology score compared to the unvaccinated group challenged with *E. coli* (*p* < 0.01) and a lower overall air sac lesion score (*p* < 0.05). Overall lung and spleen lesion scores only differed significantly between the unvaccinated *E. coli* challenged group compared to the vehicle inoculated groups. The overall gross pathology score was 2.8 and 1.95 in the unvaccinated and vaccinated *E. coli* challenge groups, respectively, whereas the vaccinated vehicle group had a score of 0.9 and the unvaccinated vehicle group a score of 1. Conclusions: A protective effect of an autogenous vaccine was found utilising an aerogenous model of colibacillosis through multiple methods of evaluation. The findings encourage the continued use of autogenous vaccines and underlines the necessity of discriminative experimental models with high predictive validity when evaluating vaccine interventions.

## 1. Introduction

In poultry, extraintestinal infections with *Escherichia coli*, collectively referred to as colibacillosis, are a major cause of mortality, impaired animal welfare, carcass downgrading, condemnations at slaughter and increase in the use of antibiotics [1]. Furthermore, public health is of concern as noticeable commonalities have been identified between avian pathogenic *E. coli* (APEC) and human extraintestinal pathogenic *E. coli* (ExPEC), which has the potential to cause severe infections [2,3]. Direct transmission of *E. coli* strains from poultry to humans has also been shown multiple times [4,5]. Therefore, there is a potential risk of an avian reservoir for hazardous *E. coli* strains emphasised by the emergence of multidrug-resistant APEC strains in poultry [6,7,8,9,10]. Effective strategies to manage colibacillosis in poultry are therefore highly warranted for numerous reasons, making especially efficient vaccines essential to limit disease development and the need for antibiotics. Despite the vast *E. coli* challenges in poultry, only a few commercial vaccines against colibacillosis are available, e.g., the live attenuated Poulvac^®^  *E. coli*. Thus, a reliance on autogenous vaccines is widespread throughout the world, but with limited knowledge of their protective properties [11]. Following an *E. coli* outbreak in Denmark during late 2014–2015, leading to an increase in antibiotic use in the broiler meat production chain with approximately 184% [12], a vaccine strategy was implemented for the first time. This strategy was based upon Poulvac^®^  *E. coli* and an autogenous vaccine containing the dominating outbreak strains [13] with the aim of protecting the broiler breeders, their progeny through vertical transmission of antibodies [14,15,16] as well as facilitating an overall reduction of APEC within the production system. Since the implementation of vaccination, attempts have been made in order to experimentally document the protective effect. However, limited protection has been shown using an ascending reproductive tract infection model [17,18]. Yet, due to a generally positive perception of the preventive effect within the field, the vaccine scheme has been upheld (Personal correspondence, anonymous). To get insight into the protective capacity of the vaccination strategy, the aim of the present study was to experimentally evaluate the effect of the autogenous *E. coli* vaccine, routinely used in Danish broiler breeders, applying a novel, discriminative aerogenous infection model of colibacillosis.

## 2. Materials and Methods

### 2.1. Animals, Housing and Experimental Design

A hundred-and-twenty Ross 308 female hens at 22 weeks of age were obtained from Blenta AB, Sweden. The animals were randomly distributed to one of six coops (8.64 m^2^) containing 20 hens each, subdivided further into different study-groups, adhering to the concepts of proper randomisation, and acclimatised for one week. Forty-seven hens were implemented in the current study distributed amongst groups as follows: *n* = 20 receiving the autogenous vaccine prior to aerosol challenge with *E. coli* (v-c), *n* = 10 animals receiving only sham-injections (not vaccinated) preceding bacterial challenge (nv-c), *n* = 10 receiving the autogenous vaccine without a subsequent bacterial challenge (sham-inoculation) (v-nc), and *n* = 7 receiving both sham-injections and sham-inoculation (nv-nc). The animals were kept in coops with wood-shaving bedding, turf dust-baths, straw, hay, shelves and perches as enrichment, hooded cat-litter boxes as laying nests, *ad libitum* access to water, a temperature ranging between 16.8 and 25.8 °C, and a 12-h light-dark cycle with a 30-min dim-phase were maintained. Feed was provided once a day and consisted of commercial wholefood for egg-laying hens (Nordsjællands Andels Grovvareforening, Helsinge, Denmark), given according to a feeding-guide for commercial broiler breeders, and supplemented with wheat- and barley kernels, sunflower seeds (Brogaarden, Lynge, Denmark), and fish meal (FF Skagen, Havnevagtvej, Denmark). Throughout the study, weight registrations were obtained weekly, and egg count was obtained daily. Prior to arrival at the animal facilities, the animals had not received any vaccines against *E. coli*. At week two and six following arrival, corresponding to 23 and 27 weeks of age, respectively, the animals received either 0.5 mL of the autogenous vaccine according to the manufacturer’s description, or an equal amount of sterile saline, injected intramuscularly into the superficial pectoral muscle, according to group affiliation. The autogenous vaccine was manufactured by Vaxxinova GmbH (Cuxhaven, Germany), on behalf of the Technical University of Denmark, and contained three *E. coli* isolates with the following sequence- and serotypes: ST117, O78:H4 (accession number LXWV00000000.1); ST117, O53:H4; ST95, O1:H7, which had all originally been isolated from cases of colibacillosis in Denmark. At each point of vaccination, a sealed bottle originating from the same batch (no.: 44-013456) maintained at 2–8 °C prior to vaccination, was used.

### 2.2. Inoculum

The clinical poultry isolate ST117 (serotype O78:H4), also included within the autogenous vaccine, was used as challenge strain (homologous challenge), and the inoculum was prepared as follows: three days prior to inoculation, the strain, stored at −80 °C, was plated onto blood agar base (BA) (Oxoid, Roskilde, Denmark) supplemented with 5% calf blood and incubated at 37 °C for approximately 24 h. A bacterial colony was then suspended into a 50 mL plastic centrifuge vials containing 10 mL Lysogeny broth (LB), mixed using a vortex, and incubated while shaken (125 rpm) for 19 h, then, 500 µL was transferred to 250 mL glass Erlenmeyer flasks containing 50 mL of LB. The suspension was then placed under conditions equal to the overnight-LB culture and incubated for precisely four hours allowing harvest of bacteria in the exponential growth phase for inoculation. Optical density measurements were performed targeting an OD of approximately 1.3 as this had proven equal to approximately 1 × 10^9^ CFU/mL. The inoculum, as well as the sterile LB for sham-inoculation, were kept on ice until aerosolisation, and 10-fold dilutions of the inoculum were performed on Lysogeny agar for CFU confirmation.

### 2.3. E. coli Aerosol-Challenge

Two weeks following the final vaccination, at 29 weeks of age, all hens were subjected to either *E. coli* aerosols or aerosolised vehicle, according to group affiliation. Inoculations were carried out as recently described [19]. Briefly, up to 10 birds were placed in the aerosol chamber measuring 91.4 × 89.6 × 200 cm (internal measurements), containing a roof-fan with a diameter of 31.8 cm running with a constant speed of approximately 2700 rpm, an inlet-system for aerosols and an outlet-system. The latter was equipped with a HEPA filter. Employing an Omron Ultrasonic Nebulizer NE-U17 nebuliser (OMRON Healthcare Co., Ltd. Kyoto, Japan), the animals were exposed to either ten minutes of aerosolised inoculum containing *E. coli* or sterile vehicle followed by five minutes of ventilation to eliminate remaining aerosols prior to evacuating the birds. The nebuliser settings were set at 10 for both airflow volume and nebulisation volume throughout the exposure of all the groups. During the collected time (15 min), the animals were held in the aerosol chamber, air-sampling were performed in duplicates using AeroCollect^®^ (Force Technology, Hørsholm, Denmark) air-samplers and temperature registrations were made continuously. Analyses of the air samples were done by quantitative PCR at the AeroCollect^®^ by FORCE Technology’s laboratory, Denmark, and the ct-value of 26 was set. Prior to any animal exposure, the procedure was performed as described above with sterile saline to obtain air samples verifying cleanliness of the system and to equalise the otherwise potential difference in humidity levels when exposing the initial group compared to the latter. The inhaled dosage was calculated as previously described [20]. Following inoculation, animal caretakers were required to change their outer layer of clothes prior to entering the coops and a boots bath containing Virkon^TM^ S were placed in front of the gate of each coop.

### 2.4. Euthanasia and Gross Pathology

Euthanasia was executed two days post inoculation based on previous experience [19] and carried out by induction of general anaesthesia through intramuscular injection of 4 mg/kg Xylazine (Nerfasin vet. 100 mg/mL, Virbac, Kolding, Denmark) and 20 mg/kg Ketamine (Ketaminol 100 mg/mL, MSD Animal Health A/S, Denmark) into musculus gastrocnemius of the left leg. Following confirmation of deep anaesthesia through lack of righting reflex, jaw- and neck-muscle tonus and complete unresponsiveness to manipulations and movement, cervical dislocation was executed using a wall-mounted, dull-edged guillotine. Necropsies were performed blinded and in a randomised order by technically trained personnel, a pathologist holding the final word in the evaluations, and all registrations were made systematically according to a predefined descriptive scheme (the full necropsy sheet is available in Table S1). An overall lesion-score was obtained by dividing the number of lesions in each group by the total number of birds within the respective group. Post-mortem bodyweight (BW) was registered using a scale with a precision of ±0.5 g (Diesella A/S, Kolding, Denmark) prior to necropsy, and the following organs were systematically weighed on an analytical balance (±0.02 g, Mettler Instrument, Greifensee, Switzerland) liver, spleen and the right lung enabling comparison of organ/BW-ratios. Bacterial swabs were collected from the visceral facies of the liver and from the spleen by penetrating a swab into the organs. In addition, the lumen of the middle part of the trachea, the right caudal thoracic air-sac, peritoneum, the mesovarium, bone marrow of the right femur, the lumen of salpinx (middle of magnum and the infundibulum-part) as well as the left lung were sampled for bacterial assessment. Swabs were collected in all cases, and sampling of the lung was performed by retracting the left lung from the bird using sterile instruments and placing it in a stomacher bag containing 1 mL of sterile 0.9% saline followed by 60 s of stomaching (Colworth Stomacher 400, Seward, UK). For histopathology, samples of the spleen, the right lung and the left caudal thoracic air sac were collected and immediately placed into 10% buffered formalin. Air sacs were collected by adhering the wall of the left caudal thoracic air sac to a Styrofoam frame using pushpins, thereby preserving the fragile structure as leniently as possible.

### 2.5. Microbiology

After bacterial sampling, the swabs were plated onto BA using the streak-plate method and placed at 37 °C for approximately 24 h. Plate-evaluation was semi-quantitatively performed by a skilled laboratory technician unfamiliar with the study-groups, as follows: <10 colonies morphologically recognised as *E. coli* = 0, 10–50 = 1 and >50 = 2. Pure growth of *E. coli* or cultures dominated by *E. coli* were considered positive. Confirmation of tentative *E. coli* isolates was conducted by PCR as previously described [21].

### 2.6. Histopathology

Following fixation, splenic and pulmonary tissue, as well as a sample of the left caudal thoracic air-sac, were embedded in paraffin and cut into 2–4 µm cross-sections and stained with haematoxylin and eosin (H & E). All processed tissues were evaluated systematically for morphological changes at 200× magnification or higher, and a semiquantitative scoring scheme was applied. The following parameters of evaluation were utilised in the assessment of the pulmonary sections: presence of oedema graded as absent = 0, present within parabronchi and in interstitial areas = 1, or as the former with additional oedema within the capillary beds = 2; inflammation was scored as absent = 0, mild = 1 if the interparabronchial septa contained noticeable amounts of heterophils and/or mononuclear inflammatory cells, moderate = 2 in case of the former but with additional presence of heterophils in parabronchial lumens and infiltration of the interatrial septa in part of the section, or marked = 3 if the former were apparent throughout the entire section. Necrosis was evaluated as absent = 0, mild = 1 in case of one to three parabronchi or secondary bronchi, or the capillary beds of an equal amount, collectively contained less than five focal areas of necrosis, moderate = 2 if four to six of the aforementioned areas contained focal necrosis or marked = 3 if the number of affected areas exceeded six. Congestion was scored as either absent = 0 or present = 1 if more than 30% of the parabronchi within the section exhibited congested vessels in the interatrial septa. The presence of plasma cells was graded as either absent = 0 or present = 1 in case of visually identifiable plasma cells within the section. Air sacs were evaluated on the presence = 1 or absence = 0 of oedema and congestion, whilst necrosis was evaluated as absent = 0, mild = 1 if ≤3 focal areas with necrotic cells were evident or marked = 2 if >3 focal areas with necrotic cells were present or large confluent areas with fibrin, necrosis and debris hereof were present. Inflammation was evaluated as absent = 0, mild = 1 in cases with a minor elevation of inflammatory cells consisting primarily of mononuclear inflammatory cells with ≤25 heterophils throughout the section, marked = 2 with >25 heterophils. Evaluation of splenic sections was performed by grading necrosis, pooling of proteinaceous material and distortion of the tissual architecture as either absent = 0 or present = 1. An overall lesion score was calculated for each evaluated organ. In all organs, the presence of bacteria was noted, and in a subset of sections with visual identification of bacteria, *E. coli* was confirmed by immunohistochemistry as previously described [22]. All evaluations were performed randomly and blinded.

### 2.7. Ethics

The study was approved by the Danish Animal Experiments Inspectorate under the Danish Ministry of Environment and Food, and all animal procedures were performed in accordance with this approval (license no. 2019-15-0201-01611) and in agreement with the Danish law regarding animal experiments and the EU directive 2010/63. The animals were monitored continuously following exposure and if clinical signs appeared intramuscular injections, into either musculus gastrocnemius or musculus pectoralis major, of 0.3 mL/hen Bupaq Vet. (Salfarm Danmark A/S, Kolding, Denmark), containing 0.3 mg/mL Buprenorfine, were provided and sustained if necessary. Preterm euthanasia was applied in cases of insufficient therapeutic effect in accordance with predefined humane endpoints, e.g., if animals presented with severe depression, dyspnoea, lethargy or anorexia.

### 2.8. Statistics

Statistical evaluations were performed using GraphPad Prism version 9.0.1 for MacOS (Graphpad Software, Inc., La Jolla, CA, USA), and all randomisation throughout the study was performed using the rand () function in Microsoft^®^ Excel for Mac version 16.52. Normality of all continuous data was assessed by the Kolmogorov-Smirnov normality test, and if the data did not follow a normal distribution, logarithmic transformation was attempted in order to achieve normality if possible. Parametric data were compared utilising a one-way analysis of variance (ANOVA) and Tukey’s multiple comparisons test for post hoc analyses. In the case of non-parametric and ordinal data, a Kruskal-Wallis test was applied with Dunn’s multiple comparisons test for post hoc analyses. All parametric data are presented with mean ± SD, whilst the median is available for ordinal data. The significance level was set at *p* < 0.05.

## 3. Results

### 3.1. Exposure Levels and Inoculation Conditions

Air samples collected during inoculation of the v-c, v-nc, nv-c and nv-nc revealed exposure levels to *E. coli* at 8.63 × 10^5^, 0, 7.3 × 10^5^ and 0 CFU/L, respectively. The estimated dose of *E. coli* was calculated as 1.18 × 10^7^ CFU received by the birds in the v-c group and 1.0 × 10^7^ CFU by those in the nv-c group. During exposure, the temperature, measured immediately in front of the aerosol chamber, ranged from 18 to 23.7 °C.

### 3.2. Clinical Signs

Throughout the study, all groups exhibited an equal weight gain without any fluctuation succeeding vaccination, nor did egg lay differ between the groups. Clinical signs following inoculation were minimal, with only one nv-c and one v-c bird requiring buprenorphine treatment due to mild depression and anorexia.

### 3.3. Gross Pathology

**Figure 1:**
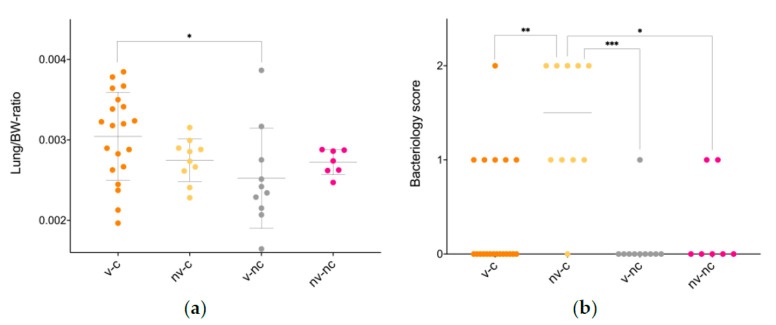
Lung/BW-ratio (**a**) and lung bacteriology (**b**). The data are presented with mean and SD or the median as appropriate, and (**a**) was analysed using a one-way ANOVA and a Tukey’s multiple comparisons test, whilst (**b**) was analysed using a Kruskal-Wallis test and Dunn’s multiple comparison test. * p < 0.05, ** p < 0.01, *** p < 0.001. Abbreviations: BW, bodyweight; nv-c, not vaccinated-challenged; nv-nc, not vaccinated-not challenged; v-nc, vaccinated-not challenged; v-c, vaccinated-challenged.

Overall, gross changes were sparse, with lesions being predominately restricted to the airways (Table 1) and included pulmonary consolidation and oedema, tracheal hyperaemia as well as thickened and opaque air sacs. Comparing the liver/BW- and the spleen/BW-ratio, no difference existed (data are available in Appendix A, Table A1), whereas a significant difference in lung/BW-ratio was present in the v-c compared to v-nc (Figure 1a). All animals, except two birds belonging to the v-c and one belonging to the v-nc, were in active lay at the time of euthanasia, defined as having either a developing egg or a fully developed egg within the oviduct.

### 3.4. Histopathology

**Figure 2:**
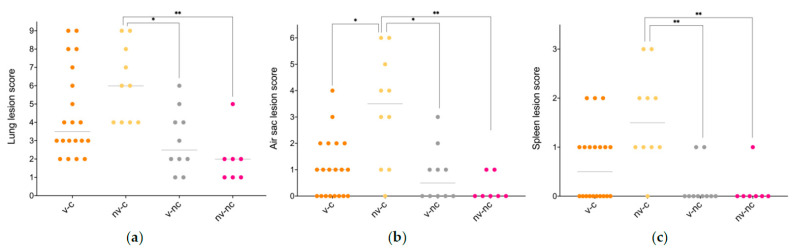
Histopathological scores of lesions in the lung, air sac and spleen. (**a**,**b**) presents the overall lung and air sac lesion score, respectively, both based upon evaluation of oedema, congestion, inflammation, and necrosis, and (**c**) exhibits the overall spleen lesion score based upon the presence of necrosis, proteinaceous fluid, and distortion of the tissue architecture. The data are presented with the median and was analysed utilising a Kruskal-Wallis test and Dunn’s multiple comparisons test. * *p* < 0.05, ** *p* < 0.01. Abbreviations: nv-c, not vaccinated-challenged; nv-nc, not vaccinated-not challenged; v-nc, vaccinated-not challenged; v-c, vaccinated-challenged.

**Figure 3:**
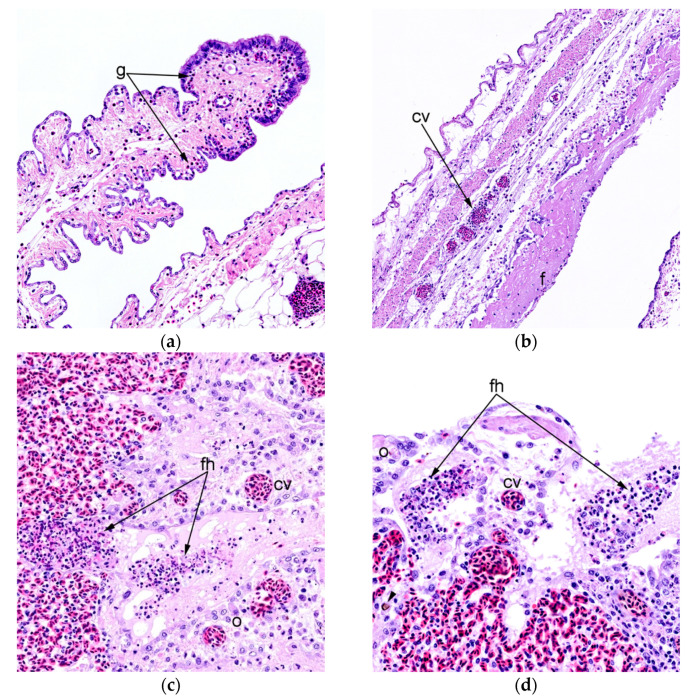
(**a**) Marked granulocytic (g) (presumed heterophils) infiltration of an air sac (**b**) Air sac with inflammation characterised by infiltration of granulocytes, most likely heterophils, as well as mononuclear inflammatory cells, congested vessels, oedema, and a layer of dense fibrinous (f) exudate on the luminal surface. A sparse number of cells embedded within the fibrinous exudate and necrotic focal areas is evident beneath the layer. (**c**) Fibrinoheterophilic (fh) exudate, cell debris and oedema (o) are present within the atria and infundibula of the parabronchus. Mild inflammation is present in the interatrial septa together with congested vessels (cv) (**d**) Similar to (**c**). A fungal fragment (arrowhead) is seen within the infundibulum. All lesions presented are from unvaccinated birds challenged with *E. coli* (positive control = nv-c group).

**Figure A1:**
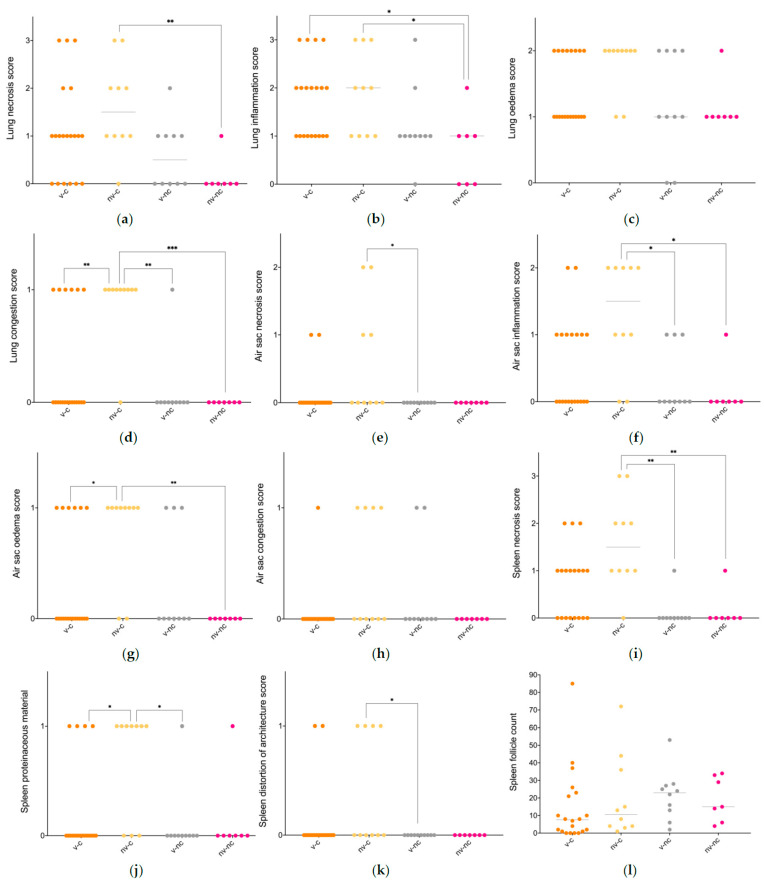
(**a**) the lung necrosis score, whilst (**b**–**d**) presents the inflammation, oedema, and congestion score of the lung, respectively. (**e**–**h**) displays the air sac necrosis, inflammation, oedema, and congestion score, respectively. (**i**) presents the spleen necrosis score, (**j**) the proteinaceous fluid score, (**k**) the tissual architecture distortion score and (**l**) the follicle count obtained from each section. Statistical analyses were performed using a Kruskal-Wallis test and Dunn’s multiple comparison test. * p < 0.05, ** p < 0.01, *** p < 0.001. Abbreviations: nv-c, not vaccinated-challenged; nv-nc, not vaccinated-not challenged; v-nc, vaccinated-not challenged; v-c, vaccinated-challenged.

Histopathological evaluation of the lung tissue revealed a significant difference in the presence of necrosis between the nv-c and the nv-nc (*p* < 0.01), the presence of inflammation between the nv-c and the nv-nc, as well as between the v-c and the nv-nc (*p* < 0.05), and in the presence of congestion between the v-c and nv-c (*p* < 0.01), the nv-c and the v-nc (*p* < 0.01) and between the nv-c and the nv-nc (*p* < 0.001). No difference was evident with respect to pulmonary oedema between any of the groups. All differences were due to the uninfected and vaccinated animals having less histopathological changes than the nv-c. The presence of plasma cells was registered in nine animals belonging to the v-c group and four animals belonging to the v-nc group and were absent in the remaining groups. Inflammation of the right thoracic air sac differed between the nv-c and the v-nc (*p* < 0.05), as well as between the nv-c and the nv-nc (*p* < 0.05) with the nv-c having higher levels of inflammation in both instances. A significant difference in oedema of the air sac was observed between the v-c and the nv-c (*p* < 0.05), and between the nv-c and the nv-nc (*p* < 0.01) with the level of oedema being higher in the nv-c birds. No difference in congestion was observed between any of the groups, whereas a significant difference in necrosis was present between the nv-c and the v-nc (*p* < 0.05). In the spleen, the level of necrosis differed between the nv-c and the v-nc, and between the nv-c and the nv-nc (*p* < 0.01 each), the degree of leaked proteaceous fluid differed between the v-c and nv-c (*p* < 0.05), and between the nv-c and the v-nc (*p* < 0.01), whilst the presence of tissual architectural distortion did not differ significantly. Figure 2 presents the overall lesion scores, and characteristic changes are displayed in Figure 3. Individual graphs on all parameters are available in Appendix B Figure A1. Regardless of group, dematiaceous fungal fragments could be identified in the pulmonary sections, primarily located in the secondary bronchi, and were consistently accompanied by an associated granulomatous inflammatory reaction.

### 3.5. Microbiology

On the day of euthanasia, all v-c, v-nc and nv-nc animals were negative for *E. coli* in the trachea, whereas three animals within the nv-c group had a tracheal score 2. Within the pulmonary samples from v-nc and nv-nc, one animal in each group exhibited positive *E. coli* growth in mixed cultures, and, in addition, one animal in nv-nc exhibited pure growth. In all these animals, the growth score was 1. In the v-c group, a single animal yielded a growth score 2, five animals a score 1, and 13 animals were negative in pulmonary samples. In contrast, only a single animal in the nv-c group exhibited a score of 0 on pulmonary samples, whereas four exhibited a growth score 1 and five a growth score 2 (Figure 1b). Positive growth from organs other than lung and trachea was few and only occurred in the birds inoculated with *E. coli*. In the v-c group, one bird had a score 1 in the mesovarium and another bird a score 2 in the peritoneum, spleen and mesovarium, as well as a score 1 in the bone marrow. One bird in the nv-c group had a growth score 1 in the peritoneum, whilst another bird yielded a score 2 in the air sacs, peritoneum, liver, spleen and mesovarium, and a score 1 in the infundibulum and bone marrow.

## 4. Discussion

A significant effect of an autogenous vaccine—routinely used within the Danish broiler breeder production—was documented in an aerogenous model of avian colibacillosis. Several disease parameters were evaluated in order to adequately compare the groups, collectively resulting in strong support of the protective properties of the vaccine. An effect, previous experimental studies have failed to demonstrate [17,18]. This inability to support the empirical confidence in the vaccine might originate in the infection model utilised, which could lack predictive validity as well as construct validity due to the degree of invasiveness. The aerosol model results in infection without the use of excessive invasiveness, and thus permits host defence mechanisms to combat the infection. Supporting this hypothesis is a previous study finding a protective effect of an autogenous *E. coli* vaccine in aerosol-inoculated brown layers [11], and a very recent study inoculating commercial pullets, vaccinated with an autogenous vaccine, intra-tracheally, also with the finding of a significant protective effect [23]. Further supporting the relevance of the use of a respiratory model is the fact that the pathogenesis of colibacillosis is believed to be aerogenous in many cases [24,25]. Bacterial growth revealed a significantly lower level of *E. coli* present in the v-c group compared to the nv-c, i.e., showing a successful elimination of bacteria within the vaccinated group. Also, histopathology revealed a lower level of lesions in the vaccinated group when comparing several organs and parameters. Lastly, the inability to statistically distinguish the v-c group from the v-nc and the nv-nc group, on multiple histopathological parameters, further supports the protective effect of the vaccine. A significant difference in lung/BW-ratio was evident between the v-c and the v-nc group in favour of the challenged group. Additionally, the remaining groups likewise exhibited a trend towards a lower ratio compared to the v-c group, however, not significant. A likely explanation of the higher lung/BW-ratio in the v-c group is related to a possibly higher activity of inflammatory processes including influx of inflammatory cells. However, histopathology was only able to show a higher level of inflammation in the v-c group compared to the nv-nc. The inability of the histopathology to reveal the cause of a tendency towards heavier lungs in the v-c group could be due to the relatively small lung sample size for histopathology. In the model, the disease outcome was relatively mild compared to previous studies [19], which is most likely related to a marked difference in the environment during the current study compared to earlier experiments as the latter was carried out at strikingly higher temperatures—a well-known risk factor for infection in poultry [26]. In the current study, a homologous challenge strain, previously determined to be highly associated with colibacillosis in Denmark [13], was evaluated. Future studies could therefore seek to investigate the immunity conferred towards a heterologous challenge.

## 5. Conclusions

In conclusion, utilising several methods for evaluation, including histopathology, microbiology and gross pathology, the protective effect of an autogenous vaccine was demonstrated in an aerogenous avian model of colibacillosis. Therefore, the use of autogenous vaccines is expected to improve animal health and aid the reduction of antibiotics, thus prompting the need for continuous utilisation of vaccines relevant to field conditions. Furthermore, the study emphasises the importance of predictive animal models when evaluating vaccine effectiveness.

## Figures and Tables

**Table 1:** An overview of the gross pathological manifestations observed.

	Vaccinated-Challenged (*n* = 20)	Not Vaccinated-Challenged (*n* = 10)	Vaccinated-Not Challenged (*n* = 10)	Not Vaccinated-Not Challenged (*n* = 7)
In-lay, euthanasia ^1^	18/20 (90%)	10/10 (100%)	9/10 (90%)	7/7 (100%)
Tracheal changes (hyperaemia, and/or mucoid, purulent and/or fibrinous tracheitis)	7/20 (35%)	5/10 (50%)	2/10 (20%)	0/7 (0%)
Pulmonary changes (consolidation, oedema and/or exudate)	13/20 (65%)	8/10 (80%)	3/10 (30%)	3/7 (42%)
Airsacculitis (hyperaemia, opaqueness and/or exudate)	10/20 (50%)	8/10 (80%)	2/10 (20%)	2/7 (28%)
Pericarditis (purulent and/or fibrinous)	0/20 (0%)	1/10 (10%)	0/10 (0%)	0/7 (0%)
Perihepatitis (purulent and/or fibrinous)	0/20 (0%)	0/10 (0%)	0/10 (0%)	0/7 (0%)
Peritonitis (purulent and/or fibrinous)	4/20 (20%)	3/10 (30%)	1/10 (10%)	1/7 (14%)
Perioophoritis (purulent and/or fibrinous)	5/20 (25%)	3/10 (30%)	1/10 (10%)	1/7 (14%)
Overall lesion score	1.95	2.8	0.9	1.0

Presented is an overview of the registrations on gross pathology obtained during necropsy. ^1^ Active lay at the time of euthanasia was defined as having either a developing egg or a fully developed egg present within the oviduct. An overall lesion score was calculated by dividing the number of recorded lesions within each group by the total number of birds within the respective group. Abbreviation: *n*, number.

## Data Availability

All data, upon which the conclusion in this study relies, are presented within the paper and the appendices.

## Supplementary Materials

The following are available online at https://www.mdpi.com/article/10.3390/vaccines9111233/s1, Table S1 Necropsy sheet.

## Appendix A

**Table A1:** Organ/BW-ratio.

	Vaccinated-Challenged (*n* = 20)	Not Vaccinated-Challenged (*n* = 10)	Vaccinated-Not Challenged (*n* = 10)	Not Vaccinated-Not Challenged (*n* = 7)
Lung/BW-ratio	3 × 10^−3^ ± 5.5 × 10^−4^ *	2.7 × 10^−3^ ± 2.7 × 10^−4^	2.5 10^−3^ ± 6.2 × 10^−4^	2.7 × 10^−3^ ± 1.6 × 10^−3^
Liver/BW-ratio	2.4 × 10^−2^ ± 3.9 × 10^−3^	2.3 × 10^−2^ ± 3.7 × 10^−3^	2.2 × 10^−2^ ± 2.8 × 10^−3^	2.3 × 10^−2^ ± 3.8 × 10^−3^
Spleen/BW-ratio	9.2 × 10^−4^ ± 1.8 × 10^−4^	8.9 × 10^−4^ ± 1.7 × 10^−4^	7.6 × 10^−4^ ± 1.6 × 10^−4^	8.2 × 10^−4^ ± 1.5 × 10^−4^

** p* < 0.05 compared to autovaccine control group. The data are presented with mean ± SD and were analysed utilising one-way ANOVA and a Tukey’s multiple comparisons test. Abbreviations: BW, bodyweight; *n*, number.
