# Supplementary material for: Protective Potential of an Autogenous Vaccine in an Aerogenous Model of Escherichia coli Infection in Broiler Breeders
Source: Vaccines (Basel). 2021 Oct 22;9(11):1233. doi: 10.3390/vaccines9111233 (PMC8624668; doi:10.3390/vaccines9111233)
Supplement: Supplementary file 1 [file vaccines-09-01233-s001.zip › vaccines-1399400-supplementary.pdf]

Necropsy sheet

Farm:		***Journal #***		***Hold #***		***Date***:	
House:		***ID #***:		***BW***:	__________________ kg	***Sex***:	F___ M___
Species:	Poultry _X_ Other ______	***Breed***:	Ross 308 __X__ Other __________________________	***Age***:	_________ weeks _____ days		
Euthanasia:	Yes_____ No_____	***Date*** *(death)*:		***Method***:	Cervical dislocation ___________ Other____________		
Comments:		***Name*** *(writer)*:					

In all organ systems, the presence of the following should be evaluated/considered as fit for individual organs: oedema / hyperaemia / haemorrhagic / exudate / ulceration / erosion / contents (e.g. intestinal) / hyperplasia / etc. If no gross pathology is observed: ***normal, iab, NC*** *or "–"should be put on record.*

Organ system/area:	***Description and/or pathoanatomical diagnosis***
Surface	
Plumage	*Normal / Ruffled / blood-smudged / naked areas*
Skin	*Intact / lesion(s) / laceration ("hanetråd" y / n) / dark coloration (head area)*
Foot pads	*Normal / discolouration: ____ mm / hyperkeratosis ___ mm /ulceration ____ mm / profundal swelling*
Natural orifices	*NC / urate (peri-cloacal) / faecal / blood / other*
Mucus membranes	*Normal / dry / hyperaemia / ulceration / dark coloration / "smudged" / other*
Eyes, conjunctiva	*Normal / hyperaemia / increased discharge / not possible to evaluate*
Other	*Obs. cyanosis / cadaverosis (mild / pronounced)*
Head and cranial "plucks"	
Sinus infraorbitalis	*Normal / hyperaemia / exudate (serous / mucopurulent / other)*
Oral cavity	*Normal / feed present / blood present / mucus present*
Esophagus	*NC / other*
Larynx	*Normal / hyperaemia / petechiae / mucus / other*
Trachea	
Lumen	*Normal / hyperaemia / haemorrhagic / mucopurulent / fibrinopurulent / catarrhalic / mucus / blood coagel / other* *Sampled:* ☐

Ingluvies (crop)<br>*Other*	*Empty / content present / filled / extended*
Corpus	
Body condition score	*Cachectic / below average / normal / above average / obese*
Hydration	*Normal / dehydrated (slightly / severely)*
Subcutis	*Normal / bursitis pre-sternalis (acute / chronic) / oedema / hyperaemia / dark coloration*
Muscles	*Normal / petechiae / ecchymoses / dark coloration / other*<br>*DPM necrosis present (sin. / dext. / bilateral) / absent*<br>*Fascial reaction present (oedema / fibrosis / haemorrhage) / absent*
Articulatio	*Art. coxae: NC / FHN (head/cartilage/collum) / other* *Art. genus: NC /other*<br>*Bonemarrow sampled:* ☐<br>*Art. intertarsalis: NC / increased synovial fluid / other*
Other	
Coelomic cavity<br>*"**Abdomen**"*	
Sternum	*Normal / fractured (# gross fractures)* *Cartilage* *mm*
Peritoneum	*NC (transparent) / milky / hyperaemia / exudate (diffuse / focally) (fibrinopurulent / serous / other)* *Sampled:* ☐
Liver	*NC / light colour / obs. steatosis / fragile / enlarged / necrosis / perihepatitis (fibrinopurulent / fibrinous) / increased texture / other* *Sampled:* ☐
Spleen	*Normal / proliferated (white pulpa pronounced) / enlarged / distended / flaccid / other* *Sampled:* ☐
Gastrointestinal tract	*Intestinum tenue: NC /* *Intestinum crassum: NC /*<br>*Caeca: NC /* *Proventriculus: NC /*<br>*Ventriculus: NC /*
Follicles	*In-lay: yes / no* *Developing egg present / fully developed egg present*<br>*Mesovarium* *Sampled:* ☐<br>*Juvenile / active follicles / flaccid (atretic) follicles / ovarian regression / hyperaemia / inactive / oophoritis (fibrinopurulent)*

Salpinx	*Juvenile / active (size) / oedema / hyperaemia / exudate / congested / inactive / pale / atrophic / other*
Infundibulum	
Magnum	*Sampled:* ☐
Isthmus	
Uterus	
Right oviduct	*Cyst / Reminiscence*
Kidneys	*Normal / swollen / increased tubular pattern / pale / other*
*"**Thorax**"*	
Pericardium	*Normal (transparent) / opaque / exudate (fibrinopurulent / fibrinous) / other*
Pericardial fat	*Normal / petechiae / hyperaemia*
Heart muscle	*NC / epicardial oedema / epicardial congestion / other*
Lungs	*Normal / congested (* $\leq$ 25% / $\approx$ 50% / $\geq$ 75% *) / pulmonary oedema (oozing) / (Suspected hypostasis) / other* *Sampled:* ☐
Saccus pneumatici	*Normal (transparent) / opaque / exudate / thickened / hyperaemia / aerosacculitis (fibrinopurulent) / other* *Sampled:* ☐
Other	

Other samples/registrations	
Pictures, measurements etc.	

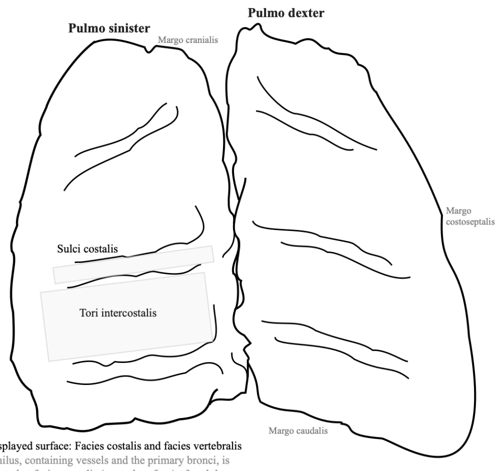

Displayed surface: Facies costalis and facies vertebralis
A hilus, containing vessels and the primary bronci, is located at facies septalis (ventral surface) of each lung
